# Simultaneous SPECT imaging of multi-targets to assist in identifying hepatic lesions

**DOI:** 10.1038/srep28812

**Published:** 2016-07-05

**Authors:** Zhide Guo, Mengna Gao, Deliang Zhang, Yesen Li, Manli Song, Rongqiang Zhuang, Xinhui Su, Guibing Chen, Ting Liu, Pingguo Liu, Hua Wu, Jin Du, Xianzhong Zhang

**Affiliations:** 1 Center for Molecular Imaging and Translational Medicine, State Key Laboratory of Molecular Vaccinology and Molecular Diagnostics, School of Public Health, Xiamen University, Xiang’an South Rd, Xiamen 361102, China; 2 Department of Isotope, China Institute of Atomic Energy, P. O. Box 2108, Beijing 102413, China; 3 The First Affiliated Hospital of Xiamen University, Zhenhai road, Xiamen 361103, China; 4 Zhongshan Hospital Affiliated of Xiamen University, Hubin South Road, Xiamen 361004, China

## Abstract

Molecular imaging technique is an attractive tool to detect liver disease at early stage. This study aims to develop a simultaneous dual-isotope single photon emission computed tomography (SPECT)/CT imaging method to assist diagnosis of hepatic tumor and liver fibrosis. Animal models of liver fibrosis and orthotopic human hepatocellular carcinoma (HCC) were established. The tracers of ^131^I-NGA and ^99m^Tc-3P-RGD_2_ were selected to target asialoglycoprotein receptor (ASGPR) on the hepatocytes and integrin *α*_v_*β*_3_ receptor in tumor or fibrotic liver, respectively. SPECT imaging and biodistribution study were carried out to verify the feasibility and superiority. As expected, ^99m^Tc-3P-RGD_2_ had the ability to evaluate liver fibrosis and detect tumor lesions. ^131^I-NGA showed that it was effective in assessing the anatomy and function of the liver. In synchronized dual-isotope SPECT/CT imaging, clear fusion images can be got within 30 minutes for diagnosing liver fibrosis and liver cancer. This new developed imaging approach enables the acquisition of different physiological information for diagnosing liver fibrosis, liver cancer and evaluating residual functional liver volume simultaneously. So synchronized dual-isotope SPECT/CT imaging with ^99m^Tc-3P-RGD_2_ and ^131^I-NGA is an effective approach to detect liver disease, especially liver fibrosis and liver cancer.

Accurate early detection of liver disease is highly desirable for appropriate therapy^1^,^2^,^3^. The determination of residual functional liver volume, imaging of intrahepatic anatomy and precise localization of the lesions are critical before complex surgical treatment performed^4^,^5^,^6^. In addition, severe injured liver can increase the risk and complexity of surgery, which associated with high morbidity and mortality^7^. Therefore, an urgent need is to develop a technique for assessing disease progression with accurate liver function evaluation, as well as monitoring the response to therapeutics.

Currently, the invasive liver biopsy (LB) is the only one reliable approach for diagnosing and staging liver fibrosis. However, sampling errors and intra- or inter- observer variability may impact the accuracy of LB for assessing liver fibrosis^8^,^9^. Many studies have elaborated the value and application of positron emission tomography (PET) and SPECT used in liver surgery and transplantation^10^,^11^,^12^. SPECT imaging of ASGPR with radiolabeled liver-specific probes neogalactosyl albumin (NGA), galactosyl human serum albumin (GSA) or neolactosyl human serum albumin (LSA) have been used to evaluate liver function^13^,^14^,^15^,^16^. However, SPECT imaging of ASGPR could not delineate the tumor size and boundary well.

RGD (arginine-glycine-aspartic) conjugates have been reported to visualize *α*_v_*β*_3_-positive tumors specifically^17^,^18^,^19^. Recent studies showed that radiolabeled RGD not only useful for early detection of integrin *α*_v_*β*_3_-positive tumors but also for staging liver fibrosis by SPECT^8^,^20^,^21^,^22^.

In order to change the “individual soldier mode” of targeted imaging, herein we proposed a simultaneous SPECT imaging strategy for multi-targets detection. SPECT imaging with dual-probe ^131^I-NGA and ^99m^Tc-3P-RGD_2_ (Fig. 1a) were investigated to help the early diagnosis of hepatic diseases, accurate evaluation of functional status and better define of both tumor size and boundary between functional liver and lesions.

SPECT is a technique with high sensitivity and has the unique capability of imaging multiple probes labeled with different isotopes at the same time. The technetium-99 m (^99m^Tc, T_1/2_ = 6 h, Eγ = 140 keV) and iodine-131 (^131^I, T_1/2_ = 8.02 d, Eγ = 364 keV) are the most common used radionuclides. The γ-rays emitted from them could be detected simultaneously in SPECT imaging and differentiated well by counting their different gamma energy, which allowing the synchronized visualization of molecular events *in vivo* by SPECT. Here we report a feasibility study of SPECT imaging of ASGPR and hepatic lesions or liver fibrosis simultaneously with ^131^I-NGA and ^99m^Tc-3P-RGD_2_ probes, respectively^23^,^24^,^25^,^26^,^27^,^28^.

## Results

### Radiolabeling of ^99m^Tc-3P-RGD_2_ and ^131^I-NGA

**Figure 1:**
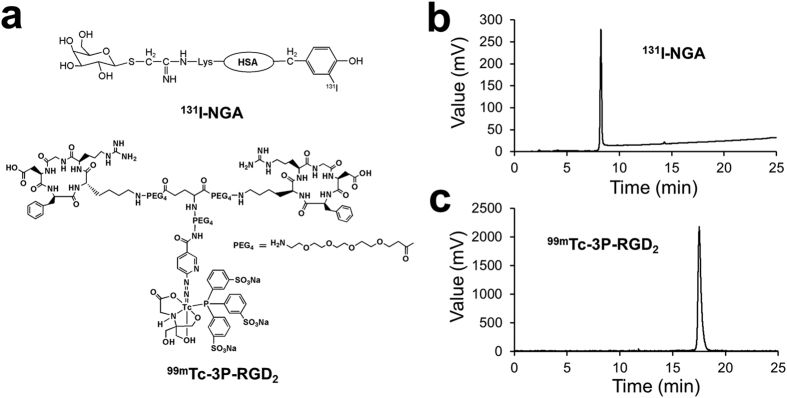
(**a**) Structure of ^131^I-NGA and ^99m^Tc-3P-RGD_2_. Radio-HPLC chromatograms of ^131^I-NGA (**b**) and ^99m^Tc-3P-RGD_2_ (**c**). The retention time of ^131^I-NGA and ^99m^Tc-3P-RGD_2_ were 8.24 and 17.49 min, respectively.

After purification, both the radiochemical purities of ^99m^Tc-3P-RGD_2_ and ^131^I-NGA were more than 95% which determined by high performance liquid chromatography (HPLC) and thin-layer chromatography (TLC) (Fig. 1b,c and Fig. S1). Their specific activities were estimated to be 66.7 TBq/mmol and 15 TBq/mmol, respectively. The resultant radiotracers were used without further purification. Radiolabeled NGA and RGD are very stable in both saline and murine serum over 4 h at 37 °C (shown in Fig. S1).

### Analyses of animal model

The liver fibrosis or tumor xenograft were verified before use. In the fibrotic model, the livers treated with CCl_4_ (Fib-4 W and Fib-8 W) showed visible fibrotic septa, while livers of normal mice are more smooth. Moreover, the tumor lesion was apparent due to the significant difference between tumor and normal hepatic tissue (Fig. S2a). As shown in Fig. S2b, higher radioactivity retention was observed in the fibrotic liver and tumor than that of normal liver in the autoradiography study, which was consistent with the biodistribution result (Supplementary Table S1). In the meantime, histopathologic change was also observed by H&E staining (Fig. S2c). Complete disruption of normal liver architecture was apparent from extensive bands of fibrous tissue and regeneration nodules. As shown in Fig. S3a,b, Sirius Red staining for control and fibrotic mice was performed as a semiquantitative assay of histological examination to determine the severity of fibrosis. Image-analysis showed that liver fibrosis progressed in a stepwise fashion in the CCl_4_ mouse model. The percentage level of red area for Fib-8 W mice (3.69 ± 0.32%) was higher than that of Fib-4 W mice (2.41 ± 0.13%) and control group (0.75 ± 0.10%). This quantification was also directly related to total collagen. While, as illustrated in Fig. S3c,d, there was remarkable increase in fibrotic liver uptake of ^18^F-FDG when compared to that of control group through PET imaging (averages of SUV from PET images were 0.68, 2.05 and 2.96 for normal mice, Fib-4 W and Fib-8 W mice, respectively). For tumor-bearing mice, the lesion was also confirmed by MRI and PET imaging (Fig. S4). The expression of integrin *α*_*v*_*β*_*3*_ in LM3 cells was confirmed by immunohistology (Fig. S5).

### *In vitro* autoradiography and histopathology

**Figure 2:**
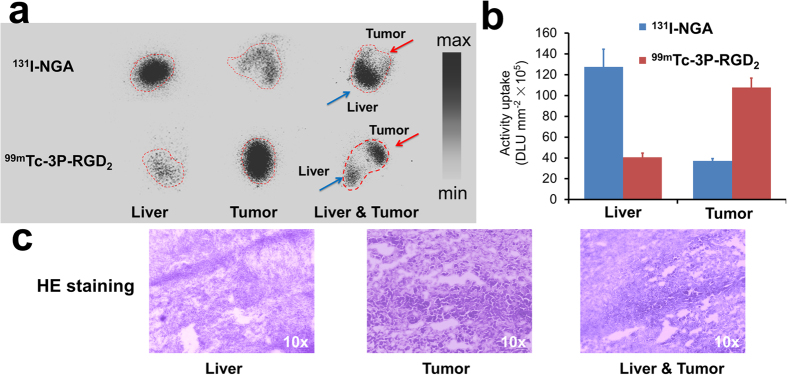
(**a**) Autoradiography of liver and tumor specimens from HCC patient. (**b**) Quantification data of autoradiographic images. (**c**) H&E staining of liver and tumor from HCC patient.

After incubated with liver specimens *in vitro*, ^131^I-NGA and ^99m^Tc-3P-RGD_2_ uptake in liver and tumor were characterized by autoradiography, respectively. ^99m^Tc-3P-RGD_2_ had higher uptake in tumor regions, while ^131^I-NGA showed lower uptake in tumor region than the surrounding hepatic tissue (Fig. 2a). Data collected from quantitative analysis further confirm these findings (Fig. 2b) and histopathologic change was also observed by H&E staining (Fig. 2c).

### MicroSPECT imaging of HCC-LM3 tumor and fibrotic mice with ^131^I-NGA only

**Figure 3:**
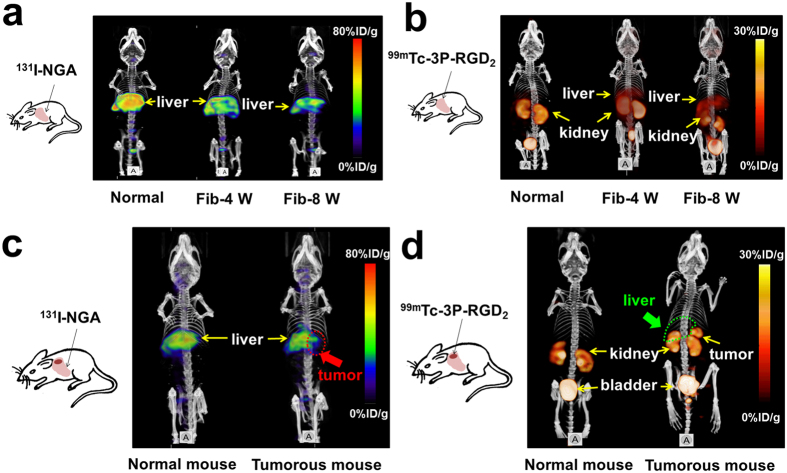
(**a**) SPECT/CT images of control and fibrotic mice at 30 min after intravenous injection of ^131^I-NGA. Highest radioactivity accumulation was found in normal liver. Fibrotic mice had a relatively low liver uptake. (**b**) SPECT/CT images of control and fibrotic mice at 30 min after intravenous injection of ^99m^Tc-3P-RGD_2_. Fibrotic mice had a relatively high liver uptake. (**c**) The SPECT/CT images of normal and LM3 tumor-bearing mice at 30 min after intravenous injection of ^131^I-NGA. High uptake in normal liver area while absent uptake in tumor location (red arrow). (**d**) The SPECT/CT images of normal and LM3 tumor-bearing mice with ^99m^Tc-3P-RGD_2_ at 30 min p.i. Tumor location could be indicated obviously and no uptake in normal liver area (green arrow). Untreated mice were used as normal controls.

**Figure 4:**
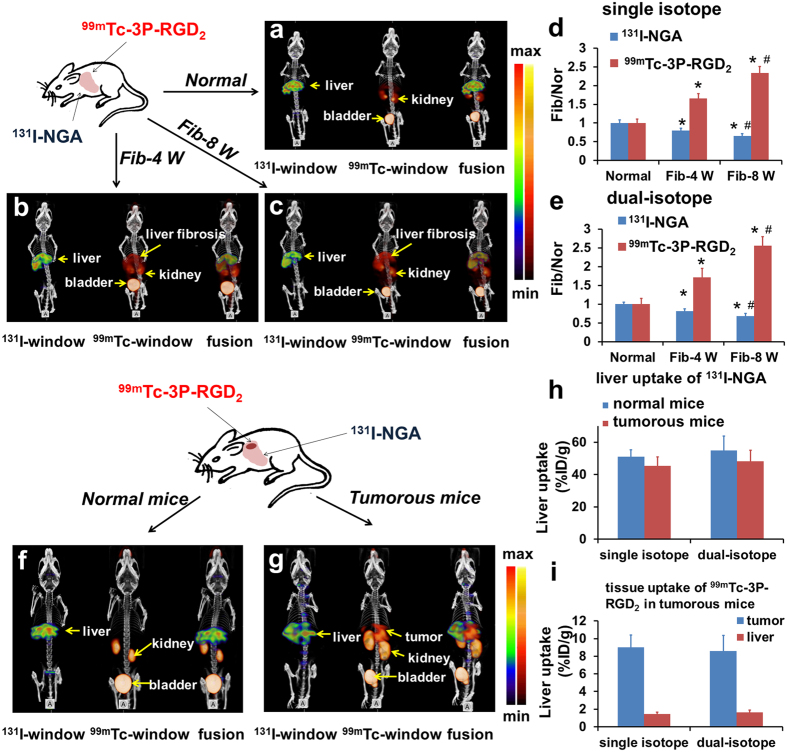
Static images of simultaneous SPECT/CT imaging at 30 min after injection of ^99m^Tc-3P-RGD_2_ and ^131^I-NGA in ^99m^Tc-window (red), ^131^I-window (green) and fusion window. Untreated mice were used as normal controls (**a,f**). For fibrotic group (**b,c**), ^99m^Tc-3P-RGD_2_ showed significant higher liver accumulation than that of normal mice at 30 min. Highest accumulation was found in 8 week fibrotic liver. While the accumulations of ^131^I-NGA in fibrotic liver much lower than that in normal liver. The ratios of Fib/Nor derived from dual-isotope imaging protocols (**e**) were consistent well with that of single isotope imaging (**d**). Liver uptake value was calculated by drawing ROIs on image and the normal liver uptake was set as 1. In the ^99m^Tc-window, for tumorous mice (**g**), activity is found in tumor and kidney. While for normal mice (**f**), no tumor lesion was found. In ^131^I-window, a surprisingly high accumulation of ^131^I-NGA in residual normal liver parenchyma was achieved. (**h**) Liver uptakes of ^131^I-NGA in normal and tumorous mice were calculated by drawing ROIs on single and dual-isotope SPECT images, respectively. (**i**) Tumor and liver uptakes of ^99m^Tc-3P-RGD_2_ in tumor bearing mice were obtained from single and dual-isotope SPECT imaging, respectively. Data were expressed as means ± SD (n = 3). In all panels, ^*^P < 0.05 versus normal mice; ^#^P < 0.05 versus FIB-4 W group.

Significant liver uptake and retention is a common feature of ASGPR targeted radiotracer. In this study, ^131^I-NGA had a significantly lower liver uptake in fibrotic mice when compared with normal mice, and the Fib-8 W mice showed the lowest liver uptake (Fig. 3a). The ratios of liver uptake in fibrotic mice to normal mice were about 0.80 (Fib-4 W) and 0.65 (Fib-8 W) at different stages, respectively (shown in Fig. 4d).

For LM3 tumor-bearing mice, the uptake of ^131^I-NGA was absented in the tumor site to show the tumor location as negative (Fig. 3c). As we know, the liver uptake of ^131^I-NGA is mediated by ASGPR, which is expressed on the surface of normal hepatic cells but not on the tumor lesion. In contrast, in the adjacent tissue of liver tumor, significant uptake of ^131^I-NGA was found due to normal ASGPR expression (Fig. 4h).

**Figure 5:**
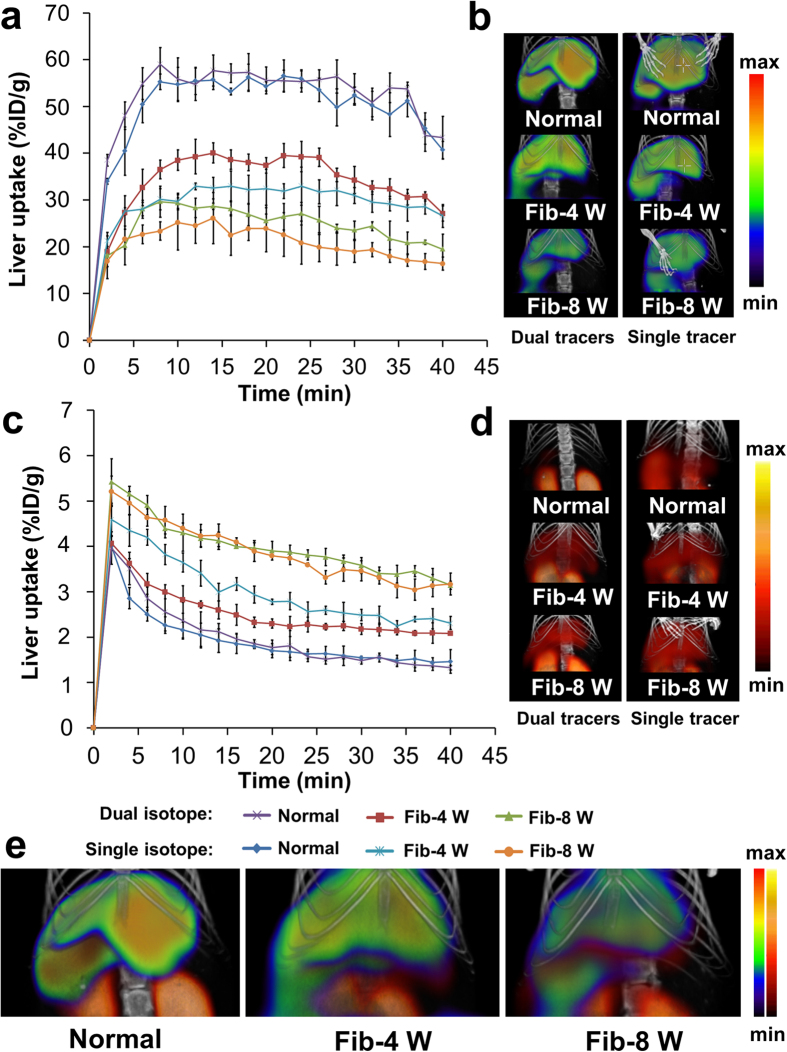
(**a**) TACs of ^131^I-NGA derived from dynamic SPECT/CT with dual and single isotope protocol, respectively. (**b**) The ^131^I-NGA images of dual and single isotope scan of control and fibrotic mice at 30 min p.i., respectively. (**c**) TACs of ^99m^Tc-3P-RGD_2_ derived from dynamic SPECT/CT scan with dual and single isotope protocol, respectively. (**d**) The ^99m^Tc-3P-RGD_2_ images of dual and single isotope scan of control and fibrotic mice at 30 min p.i., respectively. (**e**) The fusion images of ^131^I- and ^99m^Tc-windows in dual-isotope SPECT imaging.

For dynamic semiquantitative SPECT imaging, ^131^I-NGA was accumulated in liver immediately after injection. Loss uptake of ^131^I-NGA in fibrosis liver was found obviously when compared with control mouse. The ^131^I-NGA uptake value gradually decreased with the development of hepatic fibrosis (Fig. 5a,b).

### MicroSPECT imaging of HCC-LM3 tumor and fibrotic mice with ^99m^Tc-3P-RGD_2_ only

As shown in Fig. 3b, ^99m^Tc-3P-RGD_2_ had low liver uptake and high kidney uptake in the normal mice, and was excreted via the kidney to urinary bladder finally. While for the fibrotic mice, significant radioactivity was accumulated in the fibrosis liver, which might be due to the increased express of integrin *α*_v_*β*_3_ (Fig. 4d). In this study, the liver uptake ratios of fibrotic mice to normal mice were about 1.65 (Fib-4 W) and 2.34 (Fib-8 W) at different stages, respectively.

For orthotopic LM3 tumor-bearing mice, ^99m^Tc-3P-RGD_2_ showed considerable tumor uptake and low background in the healthy liver area (Fig. 3d). High activity concentrations were observed in kidney and bladder too, which consistent well with that of fibrotic mice. The tumor-to-normal liver and tumor-to-kidney ratios were calculated by regions of interests (ROIs) on SPECT/CT images (Fig. 4i, the ratios were about 6.27 and 0.87 at 30 min p.i., respectively).

For dynamic semiquantitative SPECT imaging, as shown in Fig. 5c,d, ^99m^Tc-3P-RGD_2_ showed low liver and other tissues uptake, it was mainly accumulated in kidney and excreted through renal system. The radioactivity was cleared from liver quickly in normal mouse, while for fibrotic mice, a relatively high accumulation of radioactivity was observed in the liver at early time points (at 10 min p.i.) with good retention which indicated by dynamic imaging. When compared with the Fib-4 W mice, relatively higher liver uptake was found in the Fib-8 W mice.

### Multi-targets SPECT imaging of HCC-LM3 tumor and fibrotic mice with ^99m^Tc/^131^I dual-isotope

Before *in vivo* imaging study in animals, the simulated *in vitro* experiments using phantoms of ^131^I and ^99m^Tc were performed to verify the feasibility and practicability of simultaneous dual-isotope SPECT imaging protocol (Fig. S6). The static pinhole SPECT images were acquired simultaneously at 30 min after injection of the mixture of ^99m^Tc-3P-RGD_2_ and ^131^I-NGA (Fig. 4). The tissue distribution derived from SPECT images of normal mice, fibrotic mice and orthotopic liver tumorous mice, which obtained from ^131^I-NGA window using dual-isotope protocol, almost same as the ^131^I-NGA only imaging. High liver accumulations were found in normal mice and defect liver uptake in fibrotic mice, while no uptake of lesion site in orthotopic liver tumorous mice. The correlation between radioactivity uptake and ASGPR expression indicated that it is possible to quantify the ASGPR by using dual-isotope SPECT imaging protocol to evaluate the hepatic function with ^131^I-NGA. From ^99m^Tc window, significant uptake of ^99m^Tc-3P-RGD_2_ were found in fibrotic liver due to increased expression of *α*_v_*β*_3_ on hepatic stellate cell. As shown in Fig. 4g, high tumor and kidney activity accumulation was observed in LM3 tumorous mice at 30 min p.i. Similarly, the tissue distribution pattern of ^99m^Tc-3P-RGD_2_ obtained from dual-isotope protocol was almost the same as that with single ^99m^Tc-3P-RGD_2_ tracer.

In order to further confirm the consistency of single and dual-isotope SPECT imaging, ^99m^Tc-3P-RGD_2_ or ^131^I-NGA only was injected in mice bearing LM3 tumor xenografts and liver fibrosis for SPECT imaging, respectively. The images from dual-isotope protocol have been further analyzed and compared with single isotope protocol. As demonstrated in Fig. 4d,e,h,i, the tissue distribution pattern of tracers in dual-isotope protocol was consistent well with that of single tracer. In single isotope imaging, the fibrosis-to-control ratios were about 0.80 (Fib-4 W) and 0.65 (Fib-8 W) for ^131^I-NGA, 1.65 (Fib-4 W) and 2.34 (Fib-8 W) for ^99m^Tc-3P-RGD_2_, respectively. While, in dual-isotope imaging, the ratios were about 0.81 (Fib-4 W) and 0.70 (Fib-8 W) for ^131^I-NGA, 1.71 (Fib-4 W) and 2.56 (Fib-8 W) for ^99m^Tc-3P-RGD_2_, respectively. In LM3 tumorous model (Fig. 4h,i), there is no significant difference between single isotope and dual-isotope imaging. The liver uptakes of ^131^I-NGA in tumor bearing mice were about 45.36%ID/g (single isotope) and 48.18%ID/g (dual-isotope), respectively. At the same time, the tumor uptakes of ^99m^Tc-3P-RGD_2_ were about 9.03%ID/g (single isotope) and 8.59%ID/g (dual-isotope), respectively.

Semiquantitative dynamic SPECT imaging with single isotope were also compared with that of dual-isotope protocol (Fig. 5). Based on the time-activity curves (TACs) derived from SPECT imaging with ^99m^Tc-3P-RGD_2_ and ^131^I-NGA, it was clear that both liver uptakes originated from single isotope protocol and dual isotope protocol had the same trend in normal mice and fibrotic mice, respectively. Indicated that this new developed dual-isotope imaging protocol for multi-targets has no effect on the imaging results of radiotracers.

**Figure 6:**
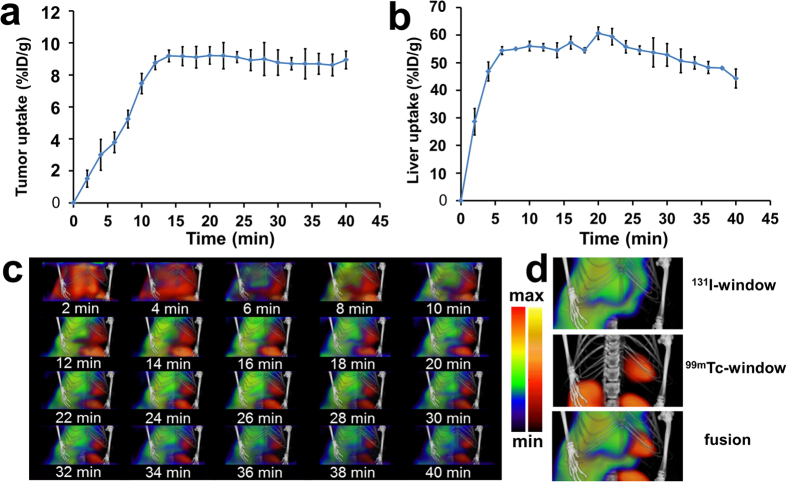
TACs derived from the dual-isotope dynamic SPECT imaging of tumor with ^99m^Tc-3P-RGD_2_ (**a**) and liver with ^131^I-NGA (**b**), respectively. (**c**) Fused SPECT images of ^99m^Tc-3P-RGD_2_ and ^131^I-NGA superimposed onto CT templates at different time points. (**d**) SPECT/CT images showed in ^131^I-window, ^99m^Tc-window and fusion window from dynamic SPECT/CT scan at 30 min after injection of ^99m^Tc-3P-RGD_2_ and ^131^I-NGA mixture.

From the TACs shown in Fig. 6, high radioactivity accumulated in the tumor lesion was observed in ^99m^Tc-3P-RGD_2_ window from 10–40 min p.i. (Fig. 6a). At the same time, the high liver accumulation was observed in ^131^I-NGA window from 5–40 min p.i. (Fig. 6b). This can be verified by the fusion SPECT images at different time points (Fig. 6c,d). In order to show the fusion effect better, 3D animation programs were presented in supporting 3D animation (tumor), which show a more intuitive structure of the tumor and liver.

## Discussion

Liver diseases exhibit complicated elements including lesion localization, acute and chronic inflammation, and changes in paracarcinomatous tissues. Usually the changes of involved receptors and microenvironment will directly affect the tumor progress and therapeutic efficacy. Here in this study, we tried to detect multiple targets simultaneously in one imaging operation to offer more biological information for disease diagnosis or therapy.

SPECT imaging for precise positioning of resection in liver surgery has attracted a lot of attention for many years. ^99m^Tc-GSA has been applied to clinical evaluation of regional liver function and estimation of postoperative hepatic functional reserve^11^,^29^. In this study, we choose the analog NGA for ^131^I-labeling and serve as ASGPR targeting probe. The other probe ^99m^Tc-3P-RGD_2_ was selected for *in situ* liver cancer detection in this study^8^,^21^. The aforesaid star probes (^131^I-NGA and ^99m^Tc-3P-RGD_2_) were adopted in this new developed imaging protocol to detect ASGPR and integrin receptors for better understanding of liver diseases. The uptake of ^131^I-NGA decreased uniformly in fibrosis liver, while nonuniform distribution with absent uptake at tumor site was observed in tumor bearing mice. From the images of ^99m^Tc-3P-RGD_2_, it had significant higher and intensive activity accumulation in tumor with clear tumor boundary, while diffuse uptake was observed in fibrotic liver. From the current data, it is possible to differentiate liver fibrosis from liver cancer based on the comprehensive information obtained by using the dual-isotope SPECT strategy. Furthermore, it will be an effective way for detection of various liver diseases and to distinguish fibrosis from liver cancer by using tumor specific tracers.

An adequate preoperative evaluation of future remnant liver (FRL) function is imperative for hepatic malignancies resection, because it is necessary to determine whether a patient can safely undergo an extended liver resection^4^,^5^,^6^. During the last decade, various tools for the virtual planning of complex liver resections, such as CT and magnetic resonance imaging (MRI), have been developed to estimate tumor size and remnant liver volume^3^,^9^,^30^,^31^. However, the volume got from CT or MRI is “anatomical volume”, not “functional volume”. Functional remnant liver volume is more valuable as it is related to liver function and complications after major liver resection. And functional liver volume (FLV) cannot be calculated by subtracting tumor volume (TuV) from total liver volume (TLV)^2^,^6^. In order to reduce the complexity of estimating volume in minimal time overhead or with minimal damage, the dual-isotope SPECT imaging could help in higher precision evaluation and better recognize of residual liver volume and liver function. In such cases, resection could be optimized to preserve a maximal amount of functional liver tissue.

Although dual-isotope SPECT imaging for myocardial perfusion has proven to be a versatile strategy in preclinical and clinical studies, a suitable dual-isotope SPECT imaging for diagnosis of hepatic lesions is still lacking and urgent^32^,^33^. To the best of our knowledge, this is the first study to use dual-isotope simultaneous SPECT imaging for diagnosis of injured liver. As a unique usage of SPECT, dual-isotope synchronized acquisition protocol has been proposed to allow imaging of different radionuclides at the same time, which reflects the comprehensive advantages. Furthermore, highly consistent scan time and exactly scan position of dual-isotope protocol resulted in satisfied image alignment of two tracers.

In summary, synchronized dual-isotope SPECT/CT imaging to assist in diagnosis of hepatic tumor and liver fibrosis is feasible, and will have great potential in clinic. This strategy offers many potential advantages: additional, valuable information content without anatomical misregistration and significantly shorten the scan time. Furthermore, this technique could reduce the burden on both patients and medical staffs.

Dual-isotope imaging has the ability to extend the application of SPECT, and with the possibility for synchronized tri-, tetra-, or more targets imaging. Although the radiation exposure of additional isotopes is an issue worthy of concern, with the continued improvement in instrument sensitivity, injected-probe activities will be reduced and the increased exposure can be alleviated.

## Methods

### Reagents and instruments

All chemicals were obtained commercially and used without further purification. Na_2_S_2_O_5_ and Chloramine-T were purchased from J&K Chemical Ltd. The eluent Na^99m^TcO_4_ and Na^131^I were obtained from Zhongshan Hospital Affiliated of Xiamen University. The liver specimens with tumor were obtained from patients with HCC (Zhongshan Hospital Affiliated of Xiamen University). The experimental methods were carried out in accordance with the relevant guidelines and all experimental protocols were approved by licensing committee of Zhongshan Hospital. In addition, the informed consent was obtained from all subjects. The HYNIC-3P-RGD_2_ kits were obtained from the First Affiliated Hospital of Xiamen University. NGA was obtained from Beijing Shihong Pharmaceutical Center of Beijing Normal University. Instant thin-layer chromatography silica gel (ITLC-SG) strips were purchased from Pall Life Sciences. The HiTrap Desalting column (filled with Sephadex G25) was purchased from GE Healthcare, eluted with phosphate buffer (PB, 0.05 mol/L, pH 7.5). Sep-Pak C18 cartridges were purchased from Waters. The ITLC strips were detected with Mini-Scan radio-TLC Scanner (BioScan, USA). The radiochemical purity was tested by Dionex Ulti-Mate 3000 HPLC (Thermo Scientific, USA) with flow-counter radioactivity detector (BioScan, USA). The radioactivity were measured with γ-counter (WIZARD 2480, Perkin-Elmer, USA) and CRC-25R Dose Calibrators (CAPIN-TEC. Inc, USA). SPECT imaging study was performed by a microSPECT/CT scanner (Mediso, HUNGARY). MRI of the tumor-bearing mice was performed by a Biospin 9.4T animal scanner (Bruker, Germany). Animal PET/CT scan was performed using Inveon device (Siemens Corp., Germany).

### Animal model

Fibrotic animal models were induced with C57BL/6 mice (female, 6 weeks). In order to induce liver fibrosis at different stages, we administered each mouse with 0.1 mL of a 20% solution of CCl_4_ in olive oil by intraperitoneal injection twice weekly for either 4 or 8 weeks. To avoid acute effects, all experiments were administered at a week after the last injection. The other twelve animals were used as controls. To establish the orthotopic HCC mouse model, each nude mice (male, 4–5 weeks) was implanted with 5 × 10^6^ human hepatocellular carcinoma HCC-LM3 cells through surgery. In the control group, animals did not experience surgery. All the mice were obtained from Laboratory Animal Center of Xiamen University. The experimental procedures and the animal use and care protocols were approved by the Institutional Animal Care and Use Committee of Xiamen University. All experimental protocols were carried out in accordance with the relevant guidelines.

### Radiolabeling and biodistribution of ^99m^Tc-3P-RGD_2_ and ^131^I-NGA

^99m^Tc-3P-RGD_2_^20^,^22^ and ^131^I-NGA^16^,^34^ were prepared and characterized according to literatures with slightly modification. The details were described in the supplementary file.

#### Analyses of animal model

All analyses protocols of animal model were carried out in accordance with the approved guidelines.

##### Biodistribution

In order to determine the binding of ^99m^Tc-3P-RGD_2_ in liver tissues during fibrosis progression, fib-4 W, fib-8 W and the control mice (n = 3 per group) were respectively injected with ^99m^Tc-3P-RGD_2_ (about 37 kBq/100 μL) via a lateral tail vein. At 30 min post-injection (p.i.), mice were sacrificed. Organs and tissues of interest were collected, weighed and counted in γ-counter. The percentage of injected dose per gram of tissue (%ID/g) was calculated. The values were expressed as mean ± SD.

##### *Ex vivo* autoradiography

After SPECT imaging with ^99m^Tc-3P-RGD_2_ at 60 min p.i., the mice were sacrificed. The livers were harvested for autoradiography study. Liver tissues were exposed to a phosphor imaging screen for one hour. After the exposure, the screen was scanned using a storage phosphor system (Cyclone Plus, PerkinElmer Instruments Inc., USA). The images were rendered at the same scale.

##### Tissue analysis

H&E and Sirius Red staining were used to assess microstructure variation. Liver tissues of fibrotic mice and tumor-bearing mice were treated with paraformaldehyde. Then paraffin-embedded liver tissue sections were stained with H&E for routine examination according to a standard procedure. Sirius Red stained sections were analyzed to score the degree of liver fibrosis^8^,^35^,^36^.

##### PET/CT and MRI

To verify fibrotic and tumor-bearing mouse model, the following PET/CT and MRI studies were performed respectively. In ^18^F-FDG PET imaging, tumor and fibrosis tissues had higher glycolytic activity and FDG uptake^37^. MRI was applied to detect the location of the tumor based on anatomical structure. All mice were anesthetized with isoflurane during the experiment.

### *In vitro* autoradiography and histopathology

These studies were carried out in accordance with the approved guidelines. The slices of human liver and tumor specimens were co-cultured *in vitro* with ^131^I-NGA and ^99m^Tc-3P-RGD_2_ for two hours at 37 °C, respectively. After incubation, the superfluous radioactivity was removed from glass slides by washing three times with cold PBS (pH 7.4, 0.2 M). The sections of tissue were harvested for autoradiography study. The autoradiographic images were quantified using OptiQuant Acquisition software to compare the ^131^I-NGA and ^99m^Tc-3P-RGD_2_ uptake (DLU mm^−2^) in different regions. The tissue sections were stained with H&E for routine examination by a skilled anatomic pathologist to arrive at the correct diagnosis.

#### MicroSPECT/CT imaging in mice

SPECT/CT imaging studies were carried out in accordance with the approved guidelines.

##### MicroSPECT/CT imaging with ^131^I-NGA only

24 mice were prepared and assigned as following groups: Control C57BL/6 mice (n = 6), 4-week fibrotic mice (Fib-4 W, n = 6) and 8-week fibrotic mice (Fib-8 W, n = 6), control nude mice (n = 3), mice with orthotopic HCC-LM3 tumor (n = 3). For SPECT imaging, each mouse was injected with 9 MBq/100 μL ^131^I-NGA through tail vein. Anesthesia was induced with isoflurane and spontaneous breathing was maintained during the scan procedure. CT data were acquired using an X-ray voltage biased to 50 kVp with a 670 μA anode current, and the projections were 720°. SPECT acquiring parameters were as follows: energy peak of 364 keV for ^131^I, window width of 20%, matrix of 256 × 256, medium zoom, and frame: 30 s. The static pinhole SPECT imaging was performed at 30 and 60 min p.i. Dynamic semiquantitative SPECT/CT imaging was performed for 40 min (20 × 120 s) after injection. TACs were derived by drawing ROIs on the SPECT/CT images. 3D images were obtained at different time point.

##### MicroSPECT/CT imaging with ^99m^Tc-3P-RGD_2_ only

SPECT imaging with ^99m^Tc-3P-RGD_2_ were performed as above. Briefly, each mouse was injected with about 18.5 MBq/100 μL ^99m^Tc-3P-RGD_2_ through tail vein. The acquiring parameters of CT and pinhole SPECT were same as above except 140 keV energy peak for ^99m^Tc. 3D iterative algorithm was used for reconstruction. Dynamic semiquantitative SPECT/CT imaging was performed for 40 min (20 × 120 s) after injection. In this protocol, scans were bound to the particular area of chest. TACs were derived by drawing ROIs on the SPECT/CT images. 3D images were obtained at different time points^21^,^38^.

##### Multi-targets SPECT/CT imaging of HCC-LM3 tumor and fibrotic mice with ^99m^Tc/^131^I dual-isotope

27 mice were prepared and assigned as following groups: Control C57BL/b6 mice (n = 6), 4-week fibrotic mice (Fib-4 W, n = 6) and 8-week fibrotic mice (Fib-8 W, n = 6), control nude mice (n = 3), mice with orthotopic HCC-LM3 tumor (n = 6). For ^99m^Tc/^131^I dual-isotope SPECT imaging, about 18.5 MBq/100 μL ^99m^Tc-3P-RGD_2_ and 9 MBq/100 μL ^131^I-NGA were mixed first and then injected into mouse through tail vein by a vein indwelling needle. The static pinhole SPECT imaging was performed at 30 and 60 min p.i. Dual-isotope imaging was enabled by different gamma photon energies of the two nuclides (^99m^Tc and ^131^I). The data were acquired simultaneously in two energy windows, thus allowing a discrimination of both isotopes (energy peak of 140 keV for ^99m^Tc and 364 keV for ^131^I, window width of 20%, matrix of 256 × 256, zoom of 1.0, and frame: 30 s). Dynamic dual-isotope semiquantitative SPECT/CT imaging was performed for 40 min after the injection of 200 μL mixed tracers. The mouse was anesthetized with isoflurane during imaging. TACs were derived by drawing ROIs in ^99m^Tc-window and ^131^I-window, respectively. The 3D images of ^99m^Tc, ^131^I and fusion were obtained at different time points^39^,^40^,^41^,^42^.

### Statistical Analysis

The collected data were presented as mean ± standard deviation (SD). Statistical analysis between groups was performed using one-way variance tests (ANOVA) followed by post-hoc tests with SPSS statistical software and P < 0.05 was considered statistically significant.

## Additional Information

**How to cite this article**: Guo, Z. *et al*. Simultaneous SPECT imaging of multi-targets to assist in identifying hepatic lesions. *Sci. Rep.* **6**, 28812; doi: 10.1038/srep28812 (2016).

## Supplementary Material

Supplementary Information

Supplementary Video
