# Supplementary material for: Simultaneous SPECT imaging of multi-targets to assist in identifying hepatic lesions
Source: Sci Rep. 2016 Jul 5;6:28812. doi: 10.1038/srep28812 (PMC4932524; doi:10.1038/srep28812)
Supplement: Supplementary Information [file srep28812-s1.doc]

**SUPPLEMENTARY INFORMATION**

**Simultaneous SPECT imaging of multi-targets to assist in identifying hepatic lesions**

Zhide Guo^1, 2^, Mengna Gao^1^, Deliang Zhang^1^, Yesen Li^1, 3^, Manli Song^1^, Rongqiang Zhuang^1^, Xinhui Su^4^, Guibing Chen^3^, Ting Liu^1^, Pingguo Liu^4^, Hua Wu^3^, Jin Du^2^, Xianzhong Zhang^1^*

^1^ Center for Molecular Imaging and Translational Medicine, State Key Laboratory of Molecular Vaccinology and Molecular Diagnostics, School of Public Health, Xiamen University, Xiang’an South Rd, Xiamen 361102, China; ^2^ Department of Isotope, China Institute of Atomic Energy, P. O. Box 2108, Beijing 102413, China; ^3^ The First Affiliated Hospital of Xiamen University, Zhenhai road, Xiamen 361103, China; ^4^ Zhongshan Hospital Affiliated of Xiamen University, Hubin South Road, Xiamen 361004, China.

Corresponding Author:

*Tel/Fax: +86-592-2880645. E-mail: zhangxzh@xmu.edu.cn (X. Z.)

**Table of Contents**

**1. Radiolabeling of ^99m^Tc-3P-RGD_2_ and ^131^I-NGA**

**2. Supplementary Table**

**Table S1.** The biodistribution result of ^99m^Tc-3P-RGD_2_ in normal mice and liver fibrosis mouse model. (%ID/g, mean ± SD, n = 3).

**3. Supplementary Figures**

- 1. **Figure S1.** The Mini SCAN results of different constituents and compounds.
  2. **Figure S2.** Photographs, autoradiographic illustrations, and H&E sections of liver tissues.
  3. **Figure S3.** Images of Sirius Red staining and PET/CT for control and fibrotic mice.
  4. **Figure S4.** MRI and PET/CT imaging of normal and tumor-bearing mice.
  5. **Figure S5.** Immunohistology results from LM3 cells.
  6. **Figure S6.** The *in vitro* simulated imaging studies using ^131^I and ^99m^Tc.
  7. **Figure S7.** The static SPECT images of normal mouse and fibrotic mice at 1 h post-injection of ^131^I-NGA.
  8. **Figure S8.** The static SPECT images of tumor mouse at 1 h post-injection of ^99m^Tc-3P-RGD_2_ and ^131^I-NGA.

**4. Supplementary Video**

The fused three-dimension (3D) animation of simultaneous ^131^I-NGA and ^99m^Tc-3P-RGD_2_ imaging in tumor bearing mice were presented in supporting 3D animation (tumor), which show a more intuitive structure of the tumor and liver.

**1 Radiolabeling and biodistribution of ^99m^Tc-3P-RGD_2_ and ^131^I-NGA**

**1.1 Preparation of ^99m^Tc-3P-RGD_2_：**About 1 mL of Na^99m^TcO_4_ (20 mCi) in saline was added into the lyophilized kit (containing 20-25 μg HYNIC-3P-RGD_2_, 5 mg TPPTS, 6.5 mg tricine, 40 mg mannitol, 38.5 mg disodium succinate hexahydrate, 12.7 mg succinic acid, and 0.1 mg pluronic acid (0.1%)).^S1^ The vial was heated at 100 ˚C for 30 min in a lead-shielded water bath and then cooled to room temperature. After labeling, a Sep-Pak C18 cartridge was used for purification procedure. The cartridge was conditioned by washing with 10 mL ethanol followed by 10 mL H_2_O, and dried with air. The radiotracer was load onto the Sep-Pak C18 cartridge and washed with 30 mL H_2_O to elute free ^99m^TcO_4_^–^ and ^99m^Tc co-ligands. After that the ^99m^Tc-3P-RGD_2_ was eluted with 80% ethanol (2 mL) and the radiochemical purity (RCP) and specific activity (SA) were tested by ITLC-SG/ACD (acid-citrate-dextrose buffer, 0.068 mol/L citrate, 0.074 mol/L glucose, pH 5.0) and radio-HPLC, respectively. The radiotracer was evaporated and diluted in PB (0.5 M phosphate buffer, pH 7.4) for further study. ^S1, S2^

**1.2 Preparation of ^131^I-NGA:** In a 1-mL vial, 1 mg NGA was dissolved in 200 μL PB (0.5 mol/L phosphate buffer, pH 7.4), followed by the addition of Na^131^I (approximately 5 mCi). Then 50 μL chloramine-T (1 mg/mL), freshly prepared in water, was added. The reaction mixture was allowed to stand for 3 min at room temperature. Then reaction was terminated by adding of 50 μL Na_2_S_2_O_5_ (2 mg/mL, freshly prepared in water). After purified by Sephadex G25, the RCP and SA of radioiodinated NGA was tested by TLC (polyamide film/saline) and HPLC, respectively (Fig. S1), then diluted in PB for further study.^S3, S4^

**1.3 Biodistribution of ^99m^Tc-3P-RGD_2_ and ^131^I-NGA were not repeated in this study. Both of the** detailed biodistribution results could be got from the following references (S2, S5-S9).

**1.4 References:**

S1: Liu, S. & Chakraborty, S. ^99m^Tc-centered one-pot synthesis for preparation of ^99m^Tc radiotracers. *Dalton T.* **40**, 6077-6086 (2011).

S2: Zhou, Y. *et al.* ^99m^Tc-labeled cyclic RGD peptides for noninvasive monitoring of tumor integrin α_v_β_3_ expression. *Mol. Imaging* **10**, 386-397 (2011).

S3: Gore, S. *et al.* Differences in the intracellular processing of the radiolabel following the uptake of iodine-125-and technetium-99m-neogalactosyl albumin by the isolated perfused rat liver. *J. Nucl. Med.* **32**, 506-511 (1991).

S4: Beppu, T. *et al.* Liver functional volumetry for portal vein embolization using a newly developed ^99m^Tc-galactosyl human serum albumin scintigraphy SPECT-computed tomography fusion system. *J. Gastroenterol.* **46**, 938-943 (2011).

S5: Sarkar, H. S. *et al*. Syntheses of several ^99m^Tc and ^131^I labeled neoglycoalbumins and their differential uptake patterns in animal biodistribution experiments. *Nucl. Med. Biol.* **22**, 589-597 (1995).

S6: Mukai, T. *et al*. Species difference in radioactivity elimination from liver parenchymal cells after injection of radiolabeled proteins. *Nucl. Med. Biol.* **26**, 281-289 (1999).

S7: Arano, Y. *et al*. Maleimidoethyl 3-(tri-n-butylstannyl)hippurate: a useful radioiodination reagent for protein radiopharmaceuticals to enhance target selective radioactivity localization. *J. Med. Chem.* **37**, 2609-2618 (1994).

S8: Yang, G. *et al*. Diversity of rgd radiotracers in monitoring antiangiogenesis of flavopiridol and paclitaxel in ovarian cancer xenograft-bearing mice. *Nucl. Med. Biol.* **41**, 856-862 (2014).

S9: Yu, X. *et al*. Small-animal spect/ct of the progression and recovery of rat liver fibrosis by using an integrin *α_v_**β_3_*-targeting radiotracer. *Radiology* **279**, 502-512 (2016).

**Supplementary Tables**

**Table S1**. The biodistribution result of ^99m^Tc-3P-RGD_2_ in normal mice and fibrotic mice. (%ID/g, mean ± SD, n = 3).

| **tissue** | **NOR** | **Fib-4 W** | **Fib-8 W** |
| --- | --- | --- | --- |
| heart | 0.81 ± 0.13 | 1.02 ± 0.21 | 0.70 ± 0.28 |
| liver | 1.29 ± 0.09 | 2.63 ± 0.34 | 3.27 ± 0.37 |
| lung | 1.27 ± 0.17 | 1.06 ± 0.14 | 1.17 ± 0.14 |
| kidney | 9.04 ± 1.92 | 9.97 ± 0.88 | 10.02 ± 1.77 |
| spleen | 1.08 ± 0.22 | 0.97 ± 0.19 | 1.08 ± 0.12 |
| stomach | 1.12 ± 0.27 | 1.02 ± 0.17 | 0.94 ± 0.18 |
| bone | 0.84 ± 0.20 | 0.62 ± 0.11 | 0.67 ± 0.08 |
| muscle | 0.51 ± 0.04 | 0.62 ± 0.11 | 0.47 ± 0.13 |
| intestines | 1.06 ±0.34 | 1.14 ± 0.23 | 1.25 ± 0.21 |
| blood | 0.21 ± 0.05 | 0.36 ± 0.08 | 0.16 ± 0.04 |

The biodistribution of ^99m^Tc-3P-RGD_2_ in fibrotic and normal mice is presented in Table S1. ^99m^Tc-3P-RGD_2_ showed significant liver accumulation at 30 min in fibrotic mice, which was higher than that of normal mice. Highest accumulation was found in liver of 8 week fibrotic mice (3.27 ± 0.37 %ID/g). That may be due to *α*_v_*β*_3_ receptor significantly upregulated on hepatic stellate cells. The kidney showed relatively high activity. The results of biodistribution indicated that ^99m^Tc-3P-RGD_2_ can be used to distinguish different stages of liver fibrosis.

**Supplementary Figures**

**
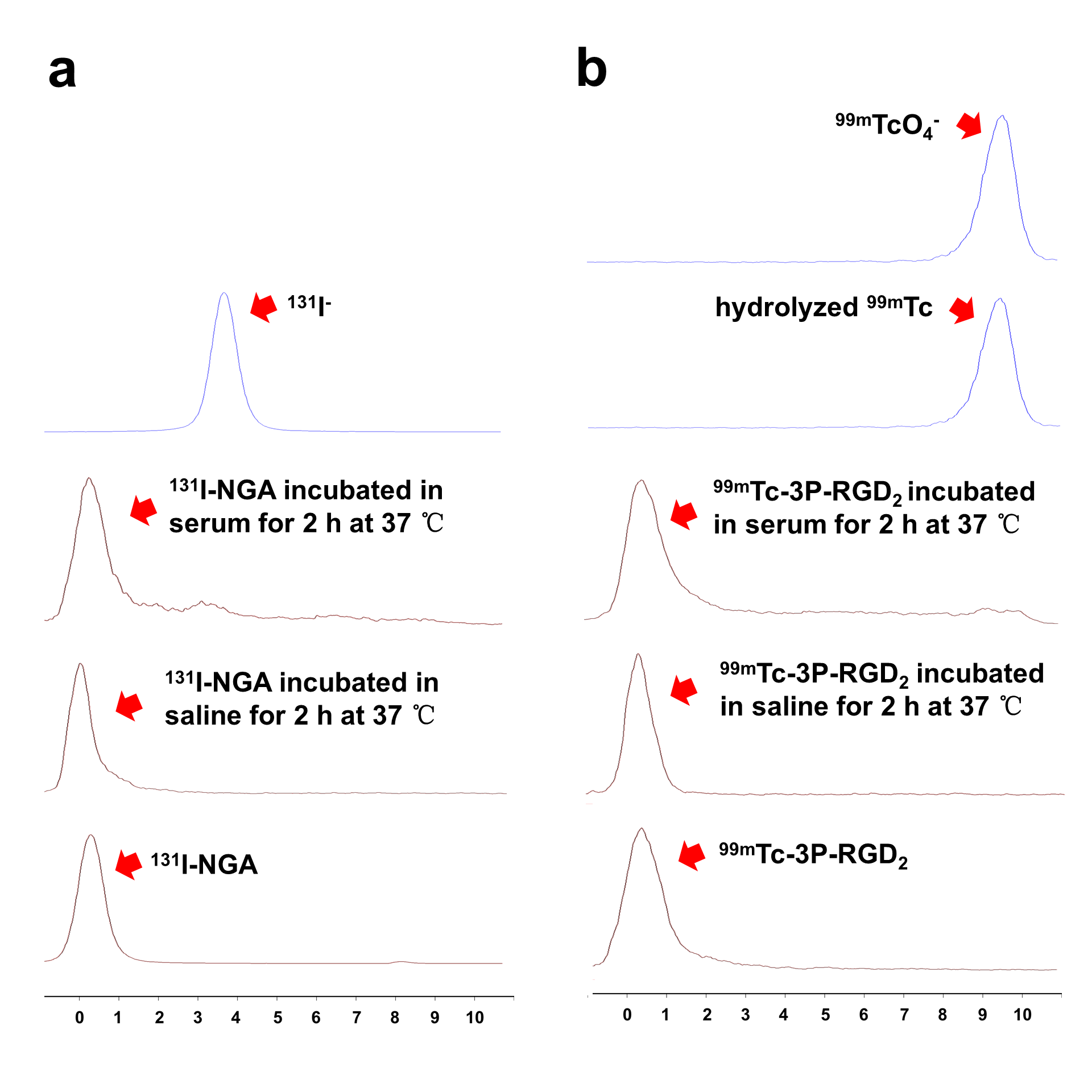
**

**Figure S1**. **The TLC results of different constituents and compounds.** (a) TLC method (polyamide film/saline) was used to identify Na^131^I and ^131^I-NGA. ^131^I-NGA stayed at the point of origin (Rf: 0-0.1), ^131^I^-^ moved at the point of middle (Rf: 0.3-0.4). (b) The labeling rate and purity of ^99m^Tc-3P-RGD_2_ were tested by ITLC-SG/ACD (acid-citrate-dextrose buffer, 0.068 mol/L citrate, 0.074 mol/L glucose, pH 5.0). ^99m^Tc-3P-RGD_2_ stayed at the point of origin (Rf: 0-0.1), whereas hydrolyzed ^99m^Tc and ^99m^TcO_4_^-^ moved at the front (Rf: 0.8-1.0). To prepare hydrolyzed ^99m^Tc, about 5 mCi/mL ^99m^TcO_4_^-^ was added to SnCl_2_ solution (4 mg/mL in 0.1 M HCl). Besides, the stability of ^131^I-NGA and ^99m^Tc-3P-RGD_2_ in saline and serum were investigated, respectively.

**
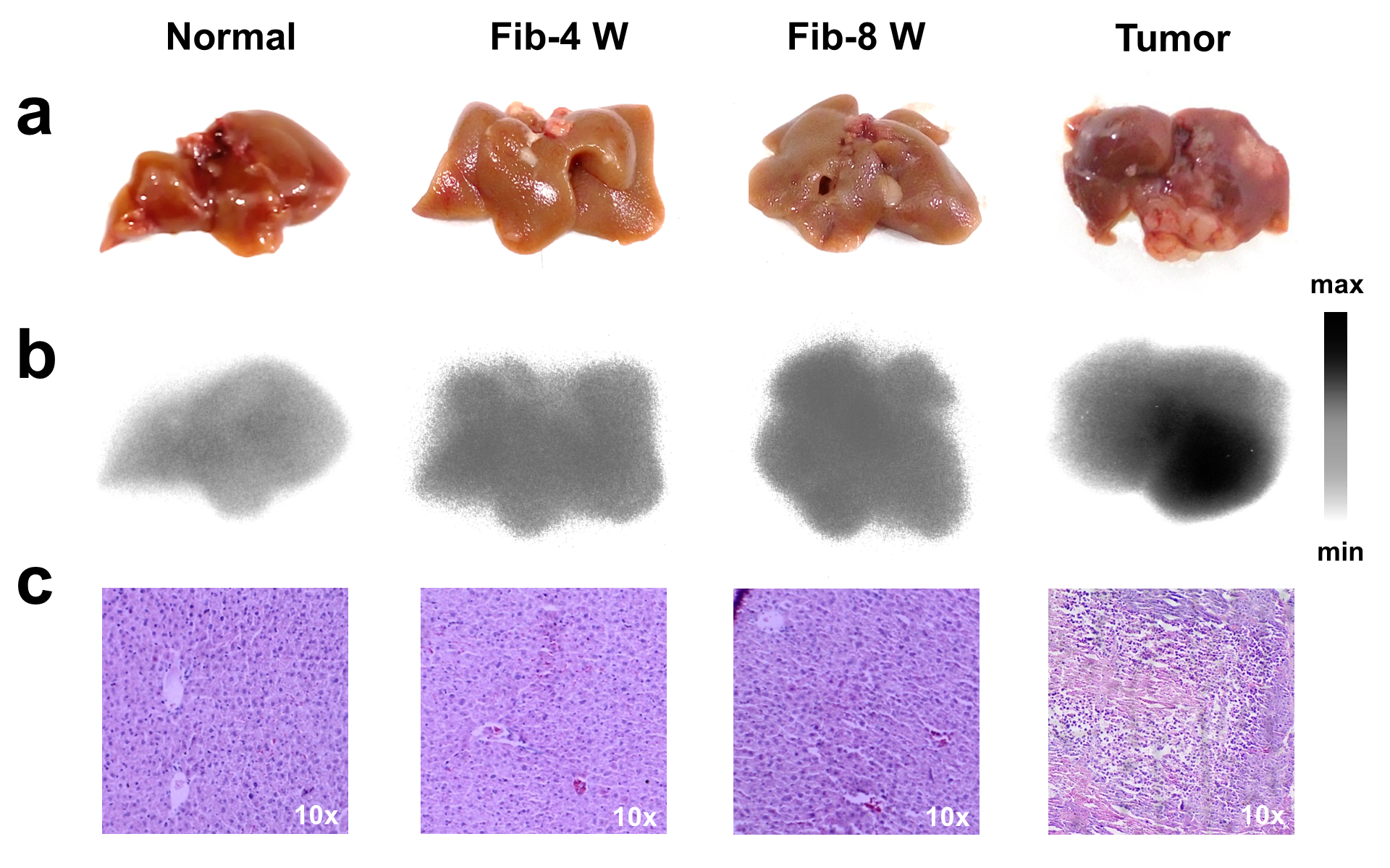
**

**Figure S2**. **Photographs, autoradiographic illustrations, and H&E sections of liver tissues.** (a) Photographs of liver tissues from normal mouse, fibrotic mouse, and LM3 tumorous mouse after dissection, respectively. (b) Autoradiographic illustration of liver tissues were acquired after SPECT imaging of 60 min for ^99m^Tc-3P-RGD_2_, respectively. (c) Representative photomicrographs of H&E sections of livers from normal mouse, fibrotic mouse, and tumor-bearing mouse, respectively.

**
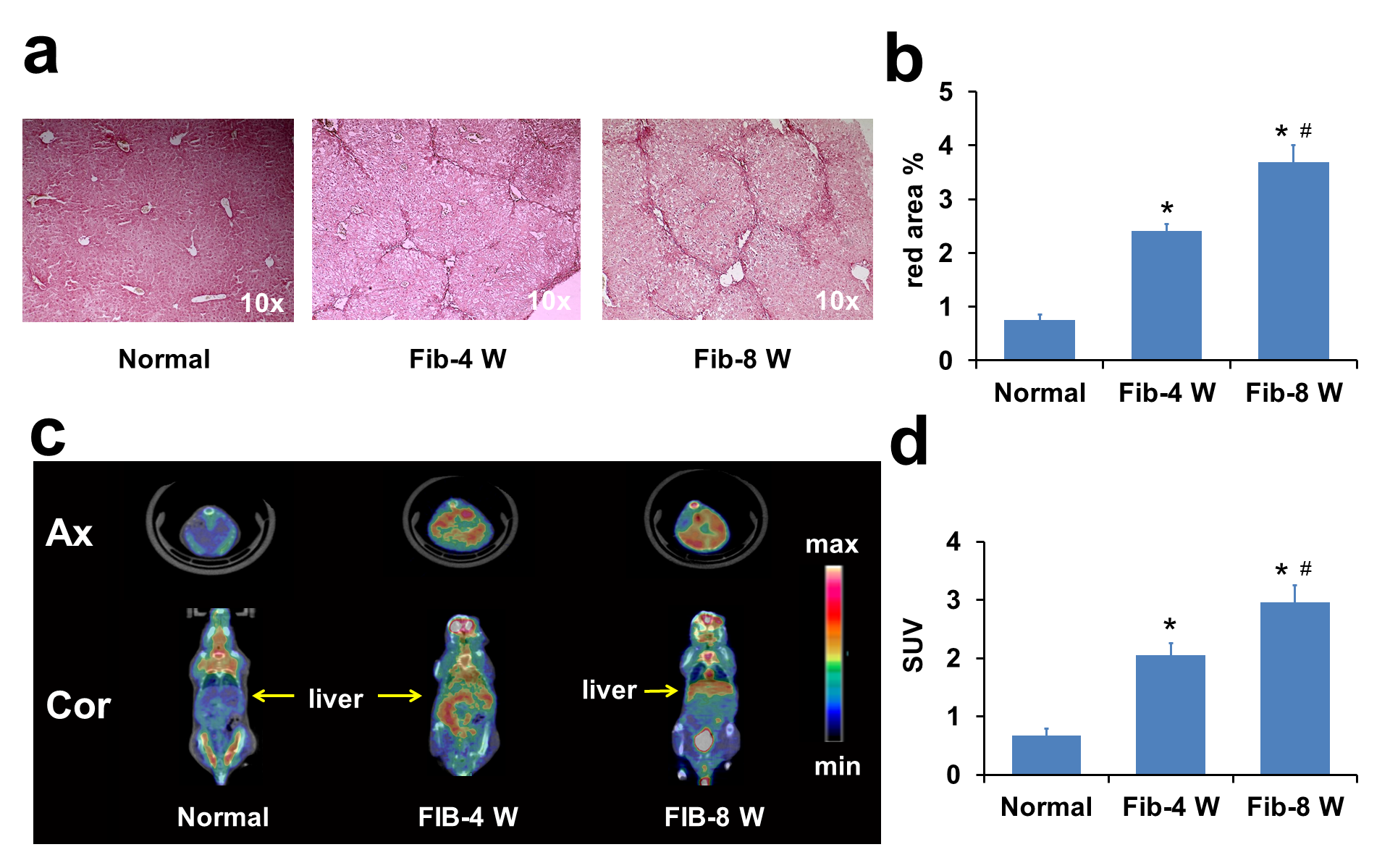
**

**Figure S3**. **Images of Sirius Red staining and PET/CT for control and fibrotic mice.** (a) Images of Sirius Red staining for control and fibrotic mice. (b) The Sirius Red stainings were quantified using Image-pro-pus software and compared to disease progression. The Sirius Red quantification is directly related to total collagen. (c) PET/CT images of normal mice, FIB-4 W and FIB-8 W mice with ^18^F-FDG, respectively. Normal mice were used as control and no lesion was found in PET images. While, there was remarkable increase in fibrotic liver uptake of ^18^F-FDG when compared to that of control group. (d) Liver uptake values of normal and fibrotic mice were calculated from the PET images. Data were expressed as means ± SD (n=3 per group). In all panels, ^*^P < 0.05 versus normal mice; ^#^P < 0.05 versus FIB-4 W group.

**
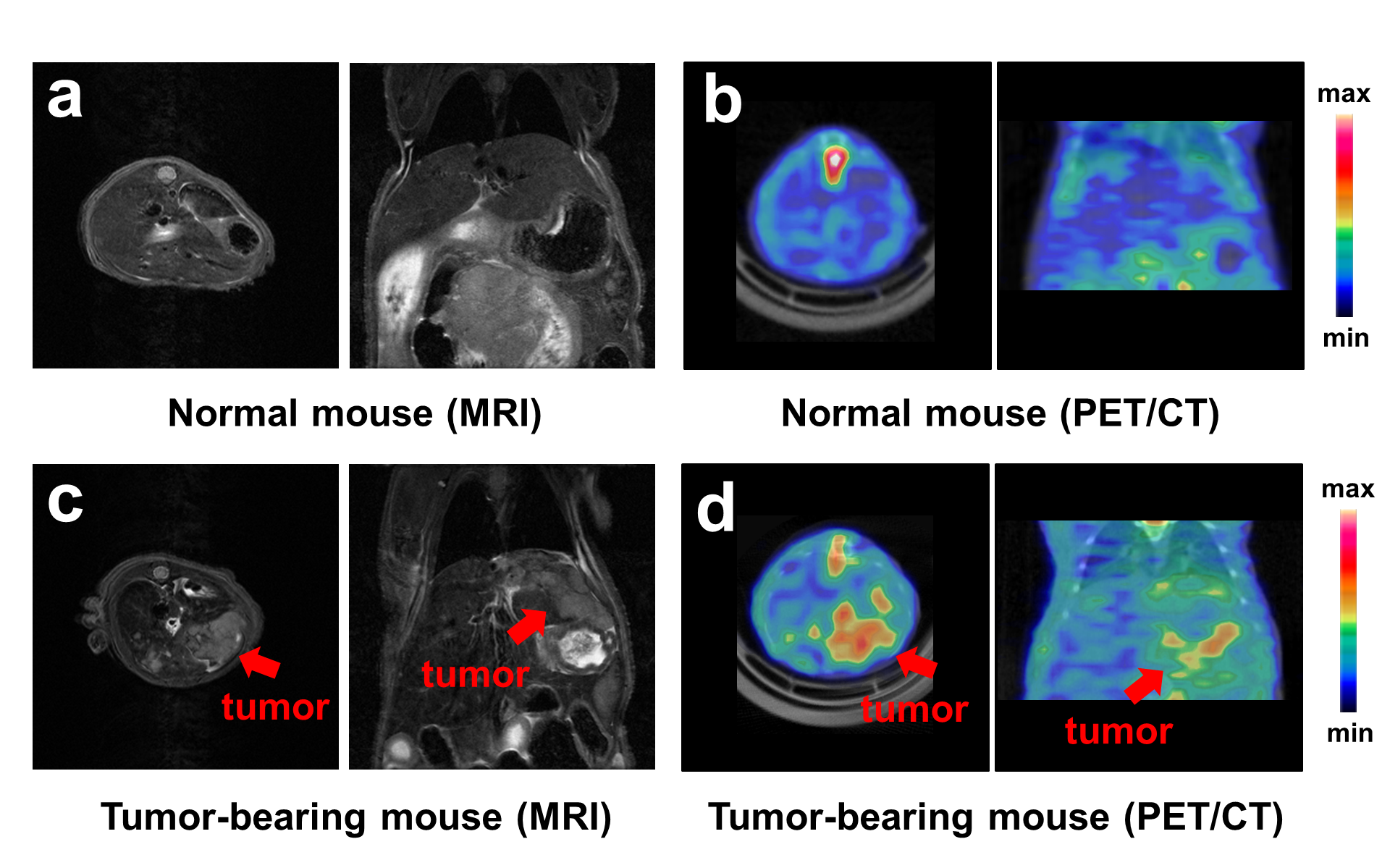
**

**Figure S4**. **MRI and PET/CT imaging of normal and tumor-bearing mice.** Normal mouse was used as control and no lesion was found through MRI (a) and ^18^F-FDG PET (b). While, for LM3 tumor-bearing mouse, the tumor lesion (indicated with red arrows) was confirmed by MRI (c) and ^18^F-FDG PET imaging (d).


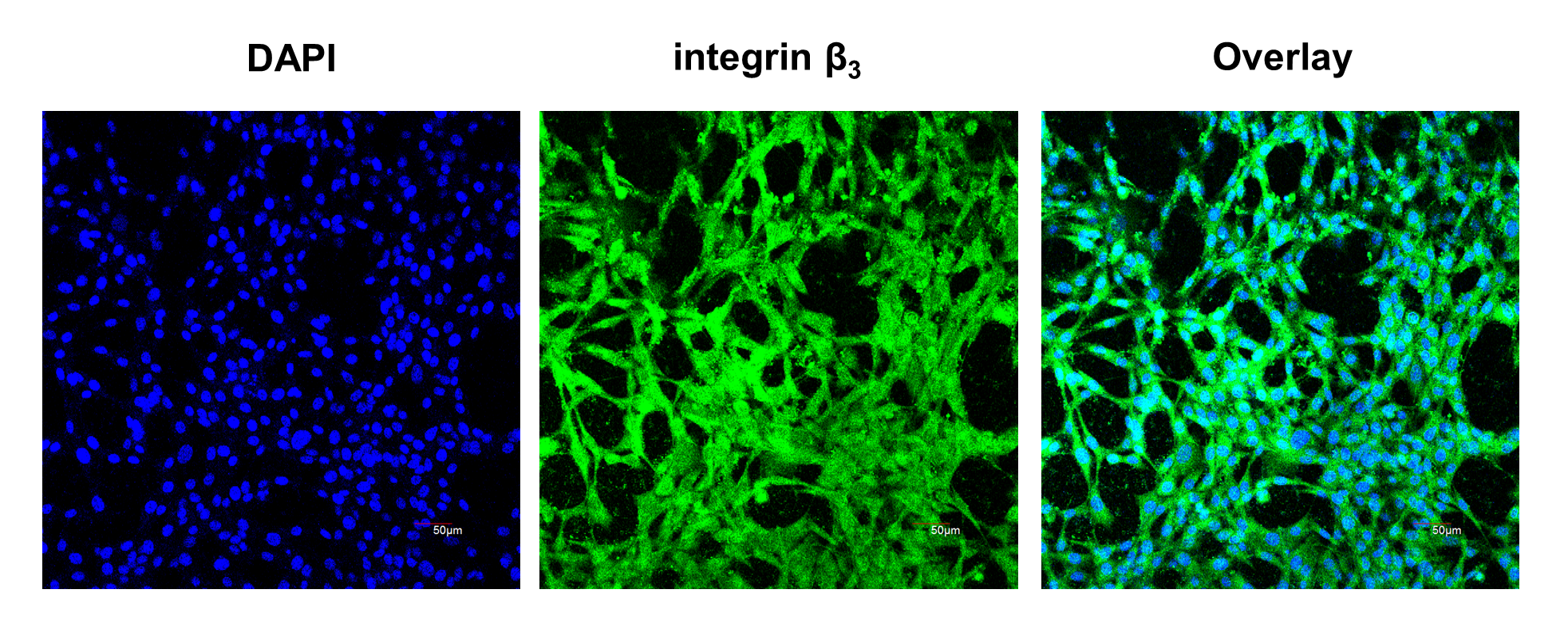


**Figure S5. Immunohistology results from LM3 cells.** Blue, DAPI; green, integrin β_3_ (FITC). Scale bar: 50 μm.


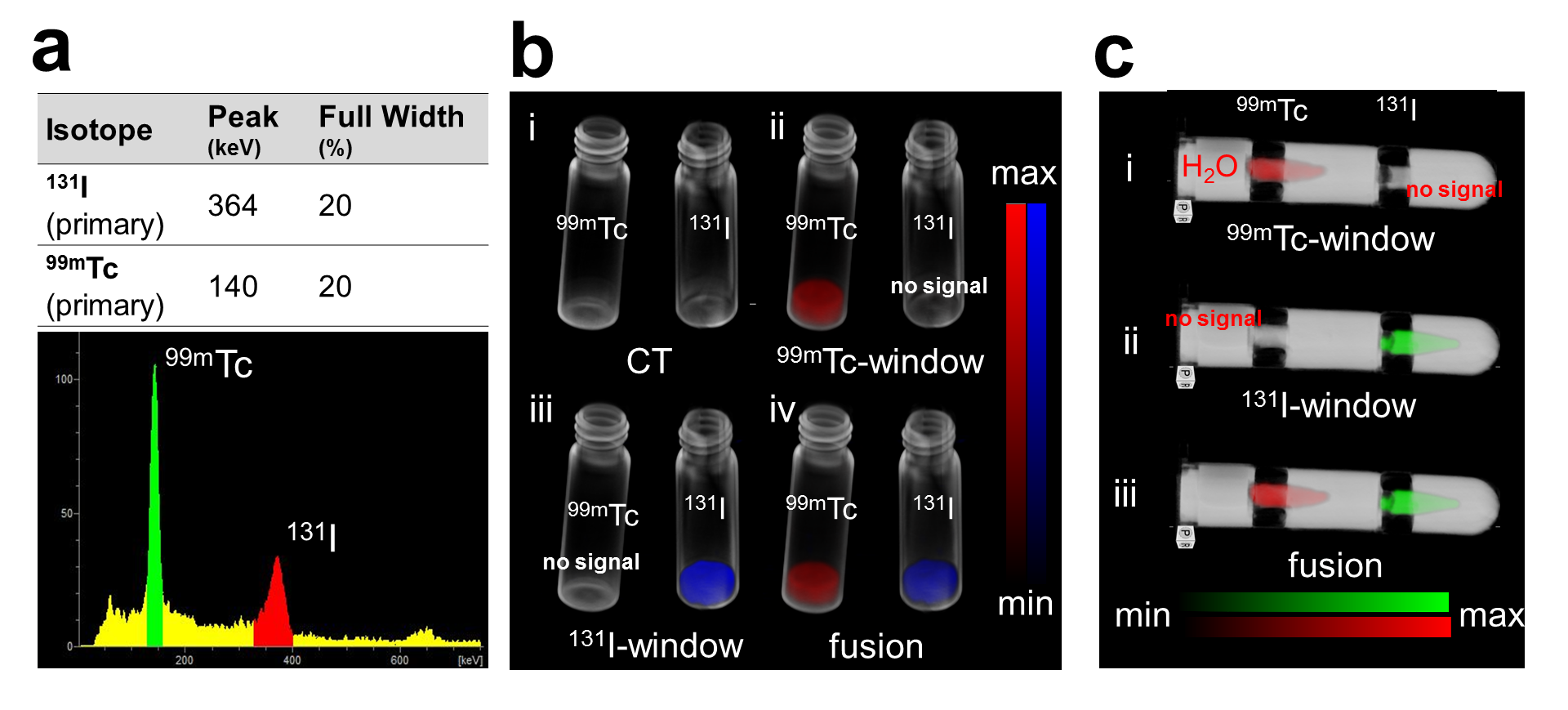


**Figure S6. The *in vitro* simulated imaging studies using ^131^I and ^99m^Tc.** (a) Different detection channels were used to distinguish the two signals based on the characteristic apex energy of radionuclide (energy peak of 140 keV for ^99m^Tc and 364 keV for ^131^I). The *in vitro* simulated imaging studies in vials (b) and centrifugal tube (c) indicate that signals do not interfere with each other.

**
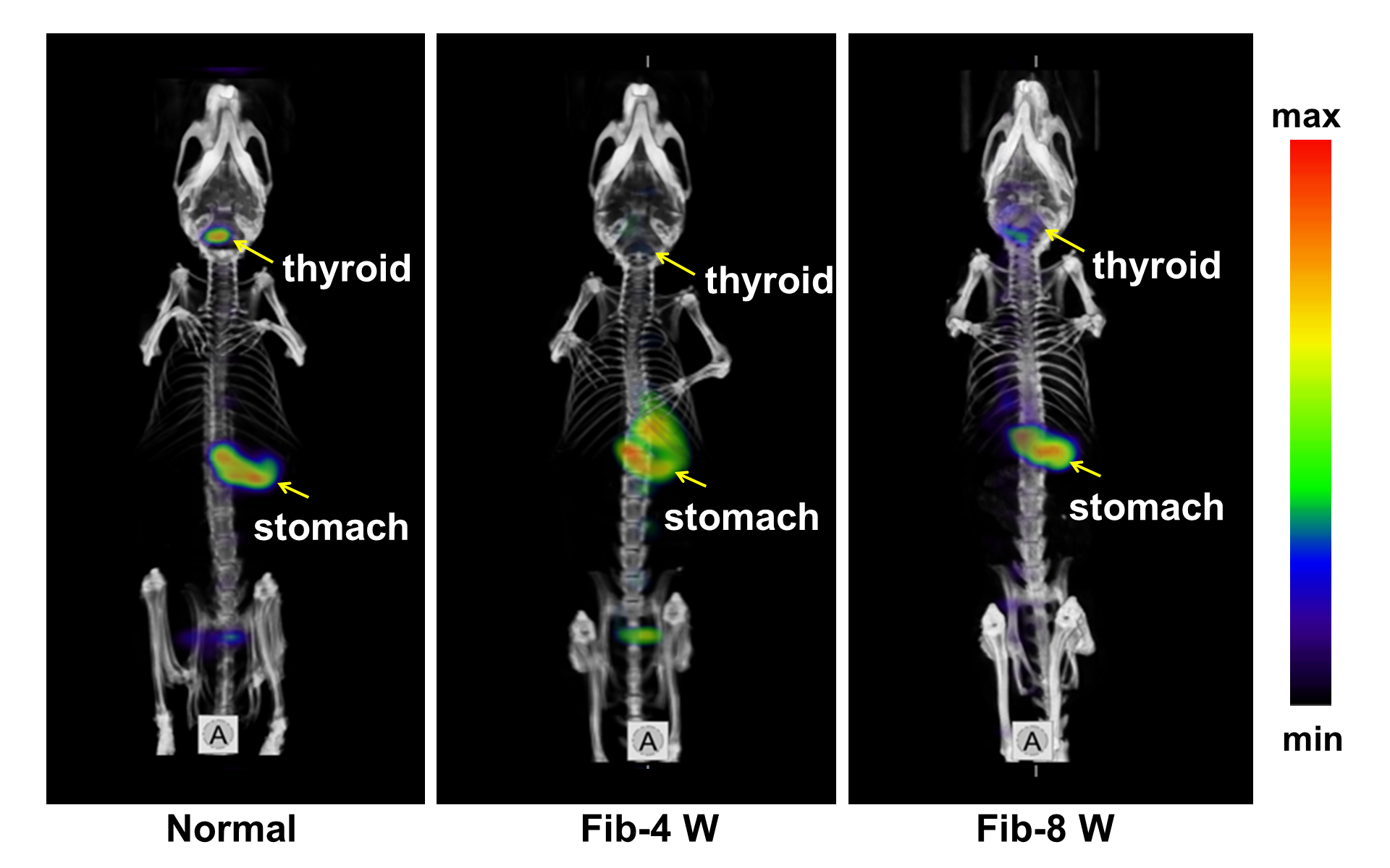
**

**Figure S7**. **The static SPECT images of normal mouse and fibrotic mice at 1 h post-injection of ^131^I-NGA.** The results showed that ^131^I-NGA exhibited high levels of radioactivity in the neck and stomach. As activity of liver was reduced at later time points, correspondingly there was a lack of a visible signal in this tissue. The data indicated that the best imaging point was in 30 minutes since injection of radiotracers.

**
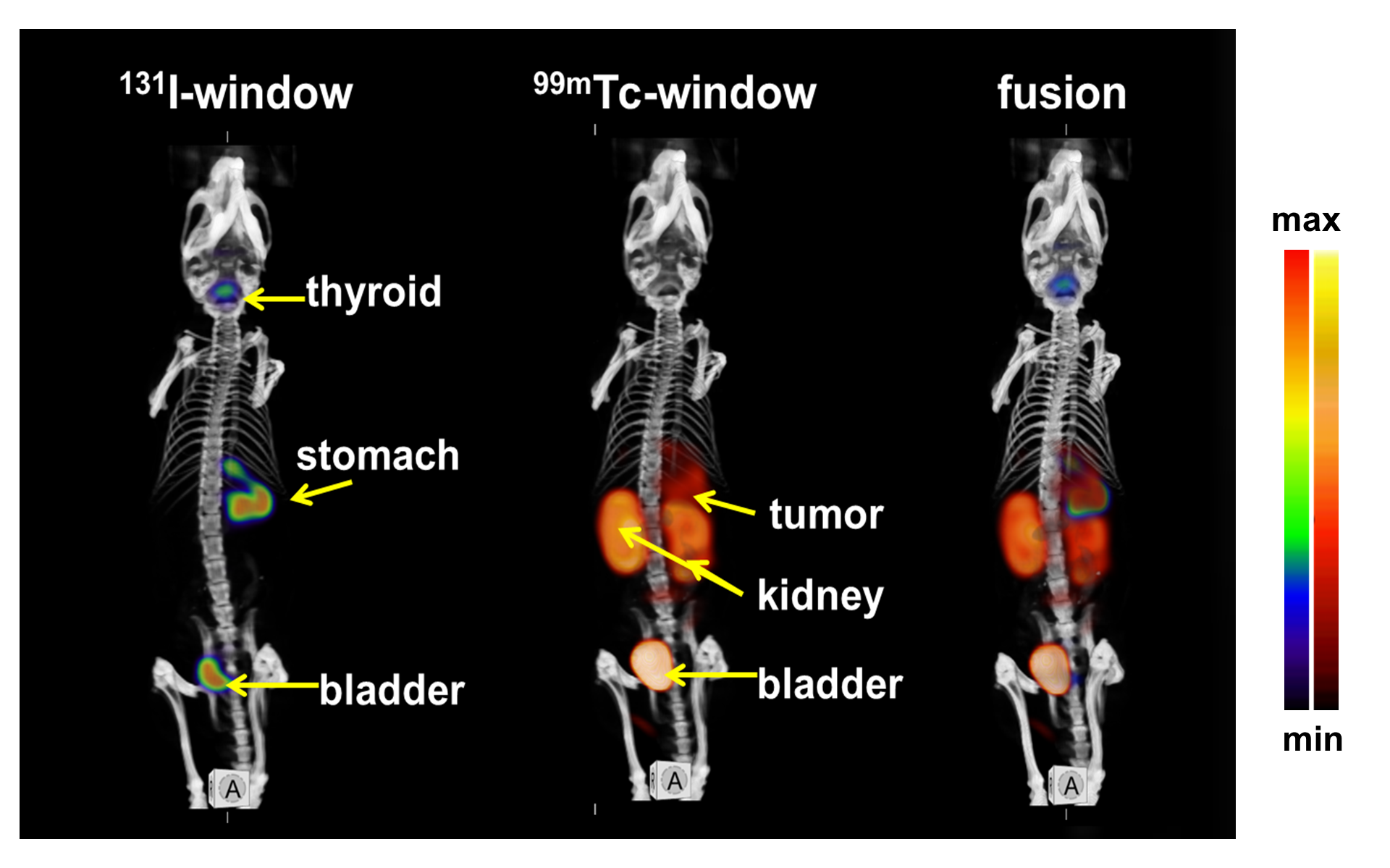
**

**Figure S8. The static SPECT images of tumorous mouse at 1 h post-injection of ^99m^Tc-3P-RGD_2_ and ^131^I-NGA.** ^131^I-NGA exhibited rapid elimination from the liver and high radioactivity levels in the stomach and neck, which was digested to ^131^I-tyrosine in hepatic lysosomes ensued with deiodination within the cytoplasm of hepatocytes. While, the ^99m^Tc-3P-RGD_2_ exhibited significant tumor uptake and retention.
